# Prevention and control measures in radiology department for COVID-19

**DOI:** 10.1007/s00330-020-06850-5

**Published:** 2020-04-16

**Authors:** Jinli Ding, Haihong Fu, Yaou Liu, Jianbo Gao, Zhenlin Li, Xin Zhao, Junhui Zheng, Wenge Sun, Hongyan Ni, Xinwu Ma, Ji Feng, Aiqin Wu, Jie Liu, Yun Wang, Pengfei Geng, Yong Chen

**Affiliations:** 1 Department of Radiology, Beijing Tiantan Hospital, Capital Medical University, Beijing, China; 2 Department of Radiology, Peking Union Medical College Hospital, Chinese Academay of Medical Sciences, Beijing, China; 3 Department of Radiology, The First Affiliated Hospital of Zhengzhou University, Zhengzhou, China; 4 Department of Radiology, West China Hospital, Sichuan University, Chengdu, China; 5 Department of Radiology, The Third Affiliated Hospital of Zhengzhou University, Zhengzhou, China; 6 Department of Radiology, Guangdong Provincial People’s Hospital, Guangzhou, China; 7 Department of Radiology, The First Affiliated Hospital of China Medical University, Shenyang, China; 8 Department of Radiology, Tianjin First Central Hospital, Tianjin, China; 9 Equipment Department, Shandong Medical Imaging Research Institute, Jinan, China; 10 Department of Radiology, Gansu Provincial Hospital, Lanzhou, China; 11 Department of Radiology, The Second Affiliated Hospital and Yuying Children’s Hospital of Wenzhou Medical University, Wenzhou, China; 12 Department of Radiology, The Third Affiliated Hospital of Zhengzhou University, Zhengzhou, China; 13 Department of Radiology, The First Hospital of Lanzhou University, Lanzhou, China

**Keywords:** Coronavirus, Pneumonia, Infection control, Radiology

## Abstract

**Abstract:**

Since a novel coronavirus was discovered from a cluster of patients with emerging pneumonia of unknown etiology in Wuhan, China, it has spread rapidly through droplet and contact transmission. Recently, the novel coronavirus pneumonia which was named COVID-19 by the World Health Organization (WHO) has been raised as a worldwide problem. Radiological examinations were confirmed as effective methods for the screening and diagnosis of COVID-19. It is reported that some radiologists and radiological technologists were infected when giving examinations to the patients with COVID-19. In order to reduce the infection risk of medical staff in radiology department, we summarized the experience on prevention and control measures in radiology department for COVID-19, aiming to guide the prevention and practical work for radiologists and radiological technologists.

**Key Points:**

*• The novel coronavirus spreads rapidly through droplet and contact transmission.*

*• Radiologists and radiological technologists were possibly infected by patients.*

*• Prevention and control measures in radiology department for COVID-19 are important.*

## Introduction

Since December 2019, a number of patients with pneumonia of unknown etiology have been reported in Wuhan, Hubei province [1]. It was reported that most of these patients worked at or lived around the local Huanan seafood wholesale market. Severe acute respiratory infection symptoms occurred in most patients, with some patients rapidly developing acute respiratory distress syndrome and acute respiratory failure [2]. On January 7, 2020, a novel type of coronavirus was identified from the throat swab sample of a patient, and was temporarily termed as 2019-nCoV by the World Health Organization (WHO). On February 11, the pneumonia caused by 2019-nCoV was formally named as COVID-19 by WHO [3].

The 2019-nCoV spreads rapidly, mainly through droplet and contact transmission. Until March 3, 2020, the number of confirmed cases reached more than 80,000 according to the report from the National Health Commission (NHC) of China. COVID-19 was classified as one of the legal class B infectious diseases in China, but taking the highest level of prevention and control measures according to the “Law of the People’s Republic of China on the Prevention and Treatment of Infectious Diseases.”

On February 11, 2020, “Diagnosis and Treatment Protocols of COVID-19 Infection (6th edition)” was published by NHC of China [4]. It indicated that X-ray and computed tomography (CT) examinations were effective methods for the screening and diagnosis of this infectious disease [5]. However, most radiologists and radiological technologists in radiology departments had less experience when facing COVID-19 patients. Examination with nonstandard prevention procedures will lead to high risks of hospital cross-infection in the radiology department. In order to effectively reduce the infection risk of radiologists and radiological technologists, a consensus on “Prevention and control measures in radiology department for COVID-19” was presented under the endeavors of many radiologists and radiological technologists led by the Chinese Society of Imaging Technology, Chinese Medical Association, aiming to guide the prevention and practical work in the radiology department.

## Personal protection levels [6]

### General protection

Wearing work clothes, disposable medical masks, work caps, and gloves (if necessary).

### Primary level of protection

It is suitable for pre-examination and triage, fever clinic, and infectious diseases clinic. Wearing disposable work caps, disposable medical masks (N95-type masks are recommended when contacting with confirmed patients), work clothes, isolation gowns, and disposable latex gloves (if necessary), and strictly implementing hand hygiene.

### Secondary level of protection

It is suitable for medical staff who has close contact with suspected or confirmed patients. Wearing disposable work caps, protective goggles or face shields (anti-fog type), medical protective masks, protective clothing or isolation gowns, disposable latex gloves, and disposable shoe covers, and strictly implementing hand hygiene.

### Third level of protection

It is suitable for aerosol-generating procedures performed for suspected or confirmed patients, such as sputum aspirating, respiratory sampling, tracheal intubation, and tracheotomy, which may cause spraying or splashing of respiratory secretions and substances of the body. Wearing disposable work caps, medical protective masks, protective face shields (full-scale respirators or positive pressure headgears are recommended), medical protective clothing, disposable latex gloves, and disposable shoe covers, and strictly implementing hand hygiene.

## Requirements for working environment and positions

### Requirements for working environment [7]

An independent medical imaging examination room is needed and different working areas should be strictly separated.

I. To prevent cross-infection, an independent medical imaging examination area or a dedicated radiological examination equipment (including X-ray photography equipment and CT scanner for infected patients) as well as a dedicated film printer should be available. According to the requirements for nosocomial infection prevention and control, the polluted area, semi-polluted area, and cleaning area need to be strictly separated and disinfected. If a dedicated examination room (such as a CT room) cannot be separated from others, strict equipment and air disinfection are required after the current patient scan and between patient encounters. Fever clinics, radiological contaminated and semi-contaminated areas, and isolation wards are the key areas for healthcare-associated infections prevention and control, which must be terminally disinfected after the examinations for confirmed patients and before the examinations for next suspected patients.
II. A dedicated radiological examinations route must be established.
III. Batch-based and time-separated services should be carried out for confirmed patients and suspected patients in fever clinic and ward, with a subsequent strict disinfection.

### Requirements for medical staff in radiology department [8]

Administrator of nosocomial infections: at least one administrator needs to be designed, who is responsible for directing and supervising the disinfection and protection in the radiology department. They must guide the whole disinfection, make a clear division, and report in time to guarantee the staff and patients by avoiding to be infected by the virus.

#### Radiographers for bedside X-ray photography

Specific radiographers need to be arranged to carry out bedside X-ray photography in key areas and take standardized photography. These radiographers are required to strictly implement the secondary level of protection. In the case of work that may cause spraying or splashing of respiratory secretions and substances of the body, such as sputum aspiration, respiratory sampling, endotracheal intubation, and tracheotomy, the third level of protection is required. After each examination, the surface of equipment should be disinfected (wiped with 75% ethanol).

#### Radiographers for digital radiography (DR) and CT examinations

The radiographers in charge of patient position should strictly implement the secondary level of protection. The radiographers in charge of operating equipment can adopt the first or secondary level of protection.

#### Staff for patient registration in the key areas

Patient registration should be completed by the radiographers in the key areas, and any material being in contact with the confirmed patients must be separately and safely stored. Staff for patient registration in the non-key area should take the primary level of prevention. It is recommended to make full use of the hospital information system (HIS), picture archiving and communication system (PACS), and radiology information system (RIS) to achieve paperless management.

#### Other staff working in the non-key areas in the radiology department

It is recommended to take the general protection when working in the cleaning areas, take the primary protection when working in the semi-contaminated areas, and take the secondary protection when working in the contaminated areas. In the case of work that may cause spraying or splashing of respiratory secretions and substances of the body, such as sputum aspiration, respiratory sampling, endotracheal intubation, and tracheotomy, the third level of protection is required.

### Working mode for radiographers in key areas (trial)

The radiographers for bedside X-ray photography, DR, and CT examinations working in the isolation area are recommended to take a 2 + 2 working mode, due to the possibility of closely contacting with the confirmed patients. In the 2 + 2 working mode, the radiographers should complete a first period of 14 workdays in the isolation area, and subsequently take a second period of 14 days in a specific dedicated isolation ward for supervised medical observation before returning to normal work.

## Processes of wearing and removing protective clothing [9, 10]

### Process of wearing protective clothing

Seven steps of hand-washing; wearing a cap; wearing a medical protective mask (taking the leak test); wearing medical protective clothing (after removing shoes); wearing latex gloves (inner layer); wearing isolation clothing; wearing latex gloves (outer layer); wearing rubber boots/shoes; wearing boot/shoe covers; wearing goggles/face shield; checking tightness.

### Process of removing protective clothing

In contaminated areas: clearing visible dirt; hand hygiene; taking off outer boot/shoe covers; hand hygiene; taking off isolation clothing and outer gloves; hand hygiene. In the semi-contaminated area: taking off goggles/mask; hand hygiene; taking off protective clothing, inner gloves, and boot covers; hand hygiene; taking off medical protective masks; taking off the cap; seven steps of hand-washing.

## Cleaning and disinfection of radiology equipment [11]

### Daily cleaning

I. Metal surface and painted surface can be wiped with soft detergent, and then dried with dry towel. Corrosive cleaning agent, corrosive detergent, and corrosive polishing agent are not allowed.
II. Chrome parts should only be wiped with a dry towel. Abrasive polishes are not allowed. Wax is suggested to protect the surface coating. Plastic surfaces can only be cleaned with soap and water.
III. Common glass cleaner except for amide products can be used to clean the touch screen. Spray the glass cleaner on the cloth or towel, and then wipe the touch screen. Be sure to remove the liquid drops in time to prevent it from flowing into the gap of the equipment.

### Equipment disinfection

I. After each examination (including X-ray photography, CT, magnetic resonance imaging, or others), the equipment must be disinfected by wiping the surface using 75% ethanol.
II. Corrosive disinfectants are not allowed.
III. Disinfectant sprays should be used carefully, as they may penetrate into the equipment, resulting in short-circuit, metal corrosion, or other damage. If the spray disinfector is needed in the room, the equipment must be shut down, cooled, and completely covered with plastic film before starting the spray disinfection. Then the equipment itself is disinfected by wiping the surface using 75% ethanol.

### Floor disinfection

Visible pollutants should firstly be removed using disposable water absorption material, and then the floor should be disinfected by using 2000 mg/L disinfectant containing chlorine (except for chlorhexidine). Furthermore, any other objects (such as handrail, door handle, window, and wall switch) should also be disinfected.

### Air disinfection

Air disinfection for the equipment room is recommended by using ultraviolet irradiation (continuous irradiation for more than 30 min), followed by ventilation for more than 30 min.

### Other disinfection

All disposable protective products must be non-reusable. The reusable protective products (such as goggles) should be placed at the designated disinfection place, and be soaked in 1000 mg/L disinfectant containing chlorine (except for chlorhexidine) or 75% ethanol for more than 1 h.

## Treatment measures of medical waste in radiology department [12]

### Collection process of medical waste

All waste from confirmed patients are regarded as infectious medical waste, and should be managed strictly according to the following process.

The process of disposing infectious waste is as follows: put the infectious waste into the medical waste collection bag (ideally be no more than ¾ full); spray the bag with 5000 mg/L disinfectant containing chlorine (except for chlorhexidine); seal the inner layer and outer layer in a Goose-Neck-Type and spray the layers with 5000 mg/L disinfectant containing chlorine (except for chlorhexidine); paste special identifications in the outer layer, and store it in the specialized site for medical wastes.

### Personnel protection

The cleaners should take the secondary level of protection and be responsible for handover registration, safe transportation, and proper storage of the infectious medical waste.

## Summary

Radiological examination, especially chest CT, plays an irreplaceable role in the diagnosis of patients with COVID-19. Radiologists, radiological technologists, and nurses must have a great command of the individual protection and disinfection procedures when working in radiology department, especially in key areas.

## Abbreviations

2019-nCoV: Novel coronavirus 2019
COVID-19: Coronavirus disease 2019
CT: Computational tomography
DR: Digital radiography
HIS: Hospital information system
NHC: National Health Commission
PACS: Picture archiving and communication system
RIS: Radiology information system
WHO: World Health Organization
